# A Single-Center Review of Palatal Fractures: Etiology, Patterns, Concomitant Injuries, and Management

**Published:** 2017-06-14

**Authors:** Ian C. Hoppe, Jordan N. Halsey, Frank S. Ciminello, Edward S. Lee, Mark S. Granick

**Affiliations:** ^a^Division of Plastic Surgery, Department of Surgery, Rutgers Biomedical Health Sciences, New Jersey Medical School, Newark; ^b^Craniofacial and Pediatric Plastic Surgery, Department of Plastic Surgery, Hackensack University Medical Center, Maywood, NJ

**Keywords:** facial bones, facial injuries, craniocerebral trauma, maxillofacial injuries, maxillary fractures

## Abstract

**Introduction:** Palatal fractures are frequently associated with facial trauma and Le Fort fractures. The complex anatomy of the midfacial skeleton makes diagnosing and treating these injuries a challenge. The goal of this study was to report our experience with the presentation, concomitant injuries, and management of palatal fractures at a level I trauma center in an urban environment. **Methods:** Data were collected for all palatal fractures diagnosed between January 2000 and December 2012 at the University Hospital in Newark, NJ. Data on patient demographics, Glasgow Coma Scale score on presentation, concomitant facial fractures, extrafacial injuries, and management strategies were collected from these records. **Results:** Of the 3147 facial fractures treated at our institution during this time period, 61 were associated with a palatal fracture following blunt trauma. There was a strong male predominance (87%) and a mean age of 35.6 years in this subset of patients. The most common causes of injury were assault and motor vehicle accident. The most common fracture patterns were alveolar, parasagittal, and para-alveolar, whereas sagittal and transverse fractures were rare. The most frequently encountered facial and extrafacial injuries were orbital fractures and intracranial hemorrhage, respectively. There was a significant association between type II sagittal fractures and traumatic brain injury (*P* < .05). **Conclusions:** Our study examines a single center's experience with palatal fractures in terms of presentation, concomitant injuries, and management strategies. Palatal fractures are most often associated with high-energy mechanisms, and the severity of injury appears to correlate with the type of palatal fracture.

Palatal fractures were first described by Rene Le Fort^1^ in his 1901 paper on maxillary fractures. These injuries continue to be found primarily in conjunction with midfacial or panfacial fractures and rarely occur in isolation. All patients with palatal fracture had an associated Le Fort I fracture in the 1998 study conducted by Hendrickson et al.^2^ Furthermore, the incidence of palatal fractures in patients with Le Fort fractures has been reported between 8% and 13.2%.^2^^-^4 Patients are typically in the second to fourth decades of life, and there is a significant male predominance.^2^^,^^5^^-^7 Children rarely experience midfacial fractures due to the elasticity of their facial skeleton and delay in synostosis of the palatal sutures.^6^^,^^8^^,^^9^ Patients with these fractures classically have a history of high-velocity impact; the most common causes of injury are motor vehicle and motorcycle accidents, whereas less common causes include assault, falls, work accidents, and firearm injury.^5^^,^^7^^,^^10^

Numerous attempts have been made to classify palatal fractures. In 1998, Hendrickson et al^2^ published a classification system based on the location and anatomical characteristics of the injury. The categories include alveolar (type I), sagittal (type II), parasagittal (type III), para-alveolar (type IV), complex (type V), and transverse (type VI) palatal fractures (Fig 1).^2^ In 2001, Park and Ock^3^ developed a classification algorithm based on the method of treatment. This paradigm was based on the presence or absence of 3 criteria: (1) the possibility of a closed reduction; (2) the site of rigid fixation; and (3) the stability of fractured segments after rigid fixation. On the basis of these criteria, fractures were assigned to one of 4 treatment groups: closed reduction (CR type); rigid fixation on the maxillary buttress, alveolar ridge, and pyriform rim (anterior or A type); rigid fixation of the palatal vault as well as anterior structures (anterior and palatal or AP type); and rigid fixation with extended immobilization (combined or C type).^3^ In 2008, Chen et al^5^ further simplified the existing classification schemes with a system based on both anatomical characteristics and optimal treatment. The categories described were sagittal (type I), transverse (type II), and comminuted (type III) fractures.^5^

The number of attempts to recategorize palatal fractures sheds light on the difficulty surgeons face when determining the approach to reconstruction. Midface fractures are complicated by the delicate bone structure, varying thickness of the soft tissue, and lack of strong sagittally oriented buttresses. Sagittal retrusion and flattening of the midface commonly occur after reconstruction due to this lack of support. The eyes and mouth functionally depend on the midface, and small misalignments can lead to enophthalmos and malocclusion.^11^ Palatal fractures are further complicated by the tendency toward malrotation and disinclination of the palatoalveolar segments.^12^

Early management of palatal fractures included wire fixation,^13^ Kirschner wires,^14^ open reduction and internal fixation with palatal splints,^15^ and transverse palatal wiring.^16^ In 1983, Manson et al^17^ described the combined use of open reduction, internal fixation, mandibulomaxillary fixation, and a palatal splint, the necessity of which was challenged several years later by Gruss and Mackinnon.^18^ In 1998, Hendrickson et al^2^ described the technique of rigid internal fixation using designer plates and screws with a limited midline split incision. Denny and Celik^4^ modified the approach to this management by recommending a palatal flap elevation in place of an incision. Later studies detailed the use of locking plates as external fixators to avoid the complications of manipulating the palatal mucoperiosteum (eg, palatal fistulae, exposure of plates and screws).^7^^,^^12^ In 2014, Ma et al^19^ described a simpler approach to external fixation using a transpalatal wire anchored by 2 screws to apply medial traction to the palatoalveolar segments.

The goal of this study was to examine a single center's experience with palatal fractures in terms of presentation, concomitant injuries, and management strategies.

## METHODS

Following institutional review board approval, data on all facial fractures occurring at a level I trauma center (University Hospital, Newark, NJ) between January 2000 and December 2012 were collected on the basis of *International Classification of Diseases, Ninth Revision* (*ICD-9*), codes. These results were further refined to include only those patients sustaining a fracture of the palate as a result of blunt trauma. Patient demographics were collected, as well as data on mechanism of injury, Glasgow Coma Scale (GCS) score on presentation, fracture locations, concomitant injuries, and fracture management strategies. Fractures were classified utilizing the scheme set forth by Hendrickson et al.^2^ SPSS (version 20; IBM) was utilized for statistical analysis. A significance value of 5% was used.

## RESULTS

**Figure 2:**
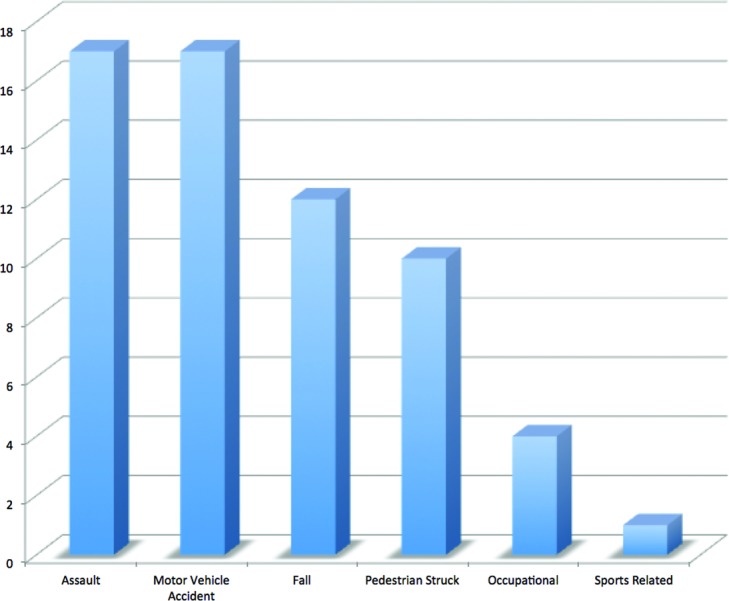
Etiology of injuries.

**Figure 3:**
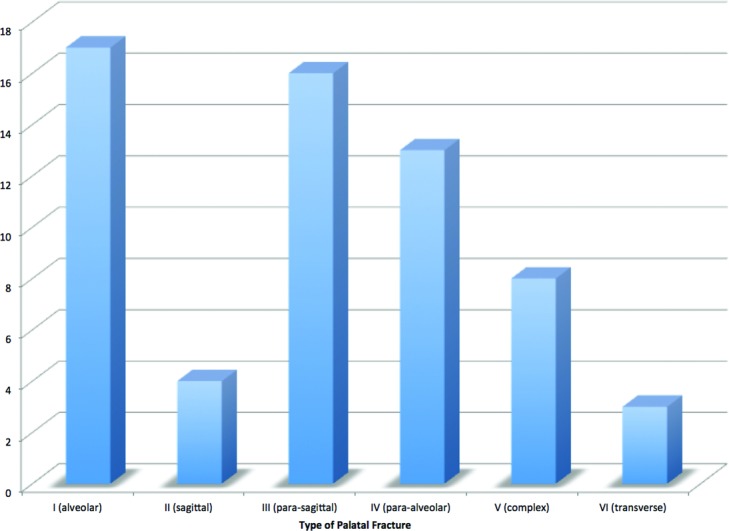
Distribution of fractures. Based on the classification from Hendrickson et al.^2^

**Figure 4:**
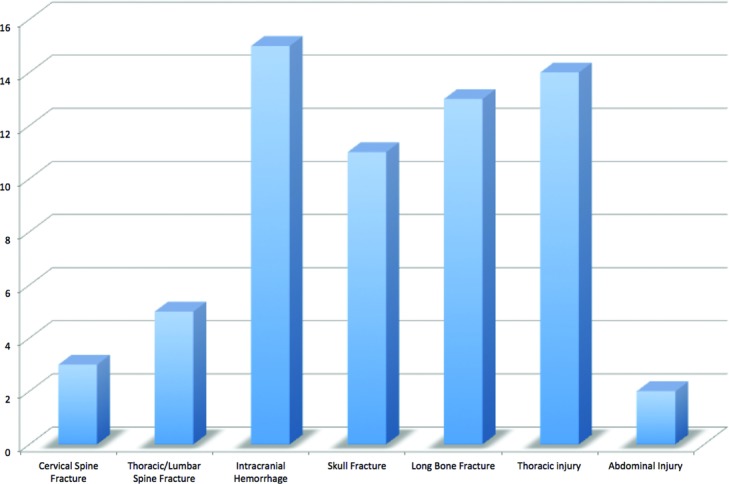
Concomitant injuries observed.

**Figure 5:**
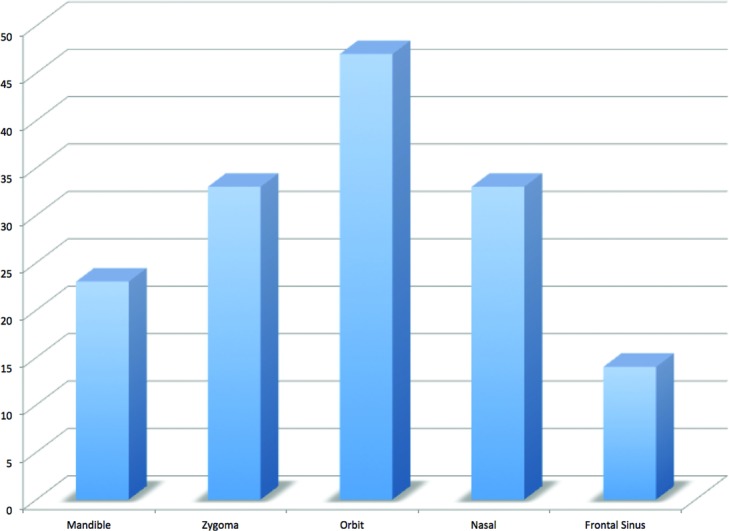
Associated fractures.

**Figure 6:**
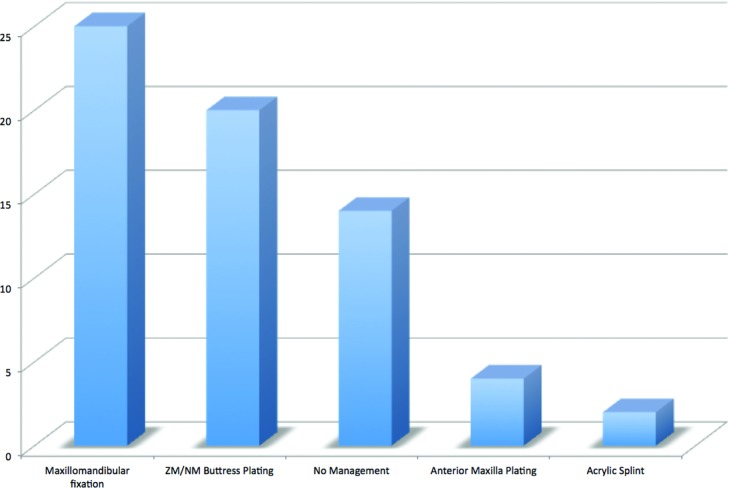
Management of palatal fractures. ZM/NM indicates zygomaticomaxillary/nasomaxillary.

During this time period, there were 3147 facial fractures treated at our institution, of which 61 were identified as a palatal fracture with an etiology of blunt trauma. There was a strong male predominance (87%) and a mean age of 35.6 years. The most common mechanisms of injury were interpersonal violence (28%) and motor vehicle accident (28%). See Figure 2. The mean GCS score on presentation was 12.4. The mean hospital length of stay was 14.5 days. The distribution of fractures is demonstrated in Figure 3. Concomitant injuries observed are shown in Figure 4. Associated fractures of the facial bones are demonstrated in Figure 5. Management strategies for the palatal fractures are shown in Figure 6. Midline splits of the palate were significantly associated with traumatic brain injury and intra-abdominal injuries (*P* < .05). There was a trend toward comminuted fractures of the palate being treated via plating of vertical maxillary buttresses and transverse fractures of the palate being associated with death (*P* > .05).

## DISCUSSION

Fractures of the palate are a fortunately rare occurrence and present a specific set of challenges in management. The choice of surgical modality depends primarily on the pattern and severity of the fracture. In our study, plating of vertical maxillary buttresses was used to treat the majority of patients with comminuted palatal fractures. Type V fractures often require a combination of stabilization techniques and therefore lack consensus with regard to management. Most authors report the need for maxillary buttress stabilization, but this alone does not provide adequate stability. The addition of intermaxillary fixation and palatal splinting provides the necessary immobilization and stability to permit bony union.^2^^,^^3^^,^^5^

In our series, the high-energy force required to fracture the palate was evidenced by the presence of numerous other concomitant injuries. In a previous study, we showed an increased incidence of intracranial hemorrhage and cervical spine fracture in pediatric patients with midface fractures compared with other facial fractures.^20^ The most common extrafacial injuries in the current study were intracranial hemorrhage, thoracic injury, long bone fracture, and skull fracture. Specifically, traumatic brain injury and intra-abdominal injury were significantly associated with midline palatal fractures in our study. Histological development of midpalatal sutures begins in adolescence and synostosis occurs between the ages of 15 and 19 years.^8^^,^^21^ While midline palatal fractures are more common in children, the strength of the synostosis makes these fractures universally rare in studies such as ours, where the mean patient age is in the fourth decade. Midline strength is likely the explanation for the low incidence of type II fractures in our study, but it may also be responsible for the significant association these fractures had with concomitant injuries. It has been suggested that the greater energy required to fracture the palate at its midline is a selective mechanism for the most severe fractures with the highest rate of associated injuries.^22^

The association between mortality and transverse palatal fracture in our series was a curious finding. To our knowledge, this fracture pattern has not been previously correlated with injury severity. Little has been reported about transverse fractures in general, as the largest palate fracture case series in the literature report only a 0% to 4.8% incidence rate and no significant associations.^2^^,^^4^^,^^5^ The tremendous energy required to fracture the palate in a transverse dimension likely portends a less favorable clinical outcome for the patient.

## CONCLUSIONS

Palatal fractures are rare injuries that occur primarily as a part of midfacial or panfacial fractures following high-energy trauma. Our study details the demographics of patients with palatal fractures at a level I trauma center over a 13-year period, as well as associations between fracture type and presentation, associated injury, and management. We report the association of midline fractures with extrafacial injury, transverse fractures with mortality, and comminuted fractures with the use of maxillary buttress fixation.

## Figures and Tables

**Figure 1:**
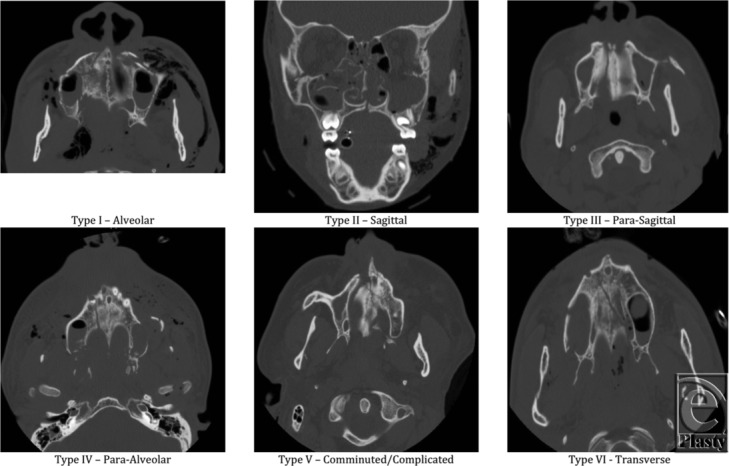
Classification of fractures. Based on the classification from Hendrickson et al.^2^
